# Generalized anxiety disorder among Bangladeshi university students during COVID-19 pandemic: gender specific findings from a cross-sectional study

**DOI:** 10.1007/s44192-022-00005-2

**Published:** 2022-02-08

**Authors:** Rasma Muzaffar, Kamrun Nahar Koly, Sabrina Choudhury, Md Abdullah Al Jubayer Biswas, Shirmin Bintay Kader, Rehnuma Abdullah, Umme Kawser, M. Tasdik Hasan, Darryn Williams, Ariful Bari Chowdhury, Helal Uddin Ahmed

**Affiliations:** 1 Department of Public Health, School of Health & Life Sciences, North South University, Dhaka, Bangladesh; 2 International Centre for Diarrhoeal Disease Research, Bangladesh (icddr,b), Dhaka, Bangladesh; 3 National Institute of Preventive and Social Medicine (NIPSOM), Dhaka, Bangladesh; 4 Department of Statistics, University of Dhaka, Dhaka, Bangladesh; 5 University of Saskatchewan, Saskatoon, Canada; 6 Department of Educational and Counselling Psychology, University of Dhaka, Dhaka, Bangladesh; 7 Department of Public Health, State University of Bangladesh, Dhaka, Bangladesh; 8 Jeeon Bangladesh Ltd., Dhaka, Bangladesh; 9 Department of Primary Care and Mental Health, University of Liverpool, Liverpool, UK; 10 London School of Hygiene and Tropical Medicine, London, UK; 11 National Institute of Mental Health, Bangladesh, Dhaka, Bangladesh

**Keywords:** Mental health & well-being, COVID-19, Anxiety, Gender, Students

## Abstract

In the current COVID-19 pandemic there are reports of deteriorating psychological conditions among university students in lower-middle-income countries (LMICs), but very little is known about the gender differences in the mental health conditions on this population. This study aims to assess generalized anxiety disorder (GAD) among university students using a gender lens during the COVID-19 pandemic. A cross-sectional study was conducted using web-based Google forms between May 2020 and August 2020 among 605 current students of two universities in Bangladesh. Within the total 605 study participants, 59.5% (360) were female. The prevalence of mild to severe anxiety disorder was 61.8% among females and 38.2% among males. In the multivariable logistic regression analysis, females were 2.21 times more likely to have anxiety compared to males [AOR: 2.21; CI 95% (1.28–53.70); p-value: 0.004] and participants’ age was negatively associated with increased levels of anxiety (AOR = 0.17; 95% CI = 0.05–0.57; *p* = 0.001). In addition, participants who were worried about academic delays were more anxious than those who were not worried about it (AOR: 2.82; 95% CI 1.50–5.31, *p* = 0.001). These findings of this study will add value to the existing limited evidence and strongly advocate in designing gender-specific, low-intensity interventions to ensure comprehensive mental health services for the young adult population of Bangladesh.

## Introduction

The coronavirus disease (COVID-19) is a global public health emergency that has been detrimental to the overall population's mental health [1]. The preventive measures for COVID-19 transmission have had a direct negative bearing on global mental health of the world population, of which one quarter are youths aged 10–24 years [2, 3]. Thus, it is unsurprising that in a lower-middle-income country like Bangladesh, COVID-19 has also affected the mental health of young adults group [4]. Evidence suggests that gender differences exist in common mental health conditions among varying age groups, in particular, generalized anxiety disorder (GAD) and depression are more prevalent among females [5]. Although gender has been shown to be an important social determinant, due consideration of this factor has been missing from the mental health burden investigations, particularly in this COVID-19 pandemic.

University students, already contending with the mental strain of tertiary educational curriculum, have faced myriad additional disruptions due to measures to curb the pandemic, such as strict lockdowns [6]. Disruptions in social support connections, usual daily routines, academic delay, and fear of COVID-19 infection have further deteriorated students’ mental health status [7]. Gender has showed a statistically significant association with mental health disorders, alongside the other major social determinants such as age, education, marital status, income level, occupation etc. [8]. Studies during the COVID-19 pandemic showed that in high-income countries, young female adults attending universities were more susceptible to common mental health conditions such as anxiety, depressive symptoms, and post-traumatic stress disorder (PTSD) [8–12]. Similarly, South Asian countries like Pakistan and India also reported a higher number of young female university students aged 18–25 years compared to men, suffering from these psychological conditions due to COVID-19 [11, 13].

Furthermore, in Bangladesh, educational institution’s pandemic-related restrictions such as lockdown have left 3.15 million university students uncertain about their future [14–16]. Students are always comfortable in interaction with their peers, teachers and enjoy the university campus atmosphere, but the lockdown has created a massive void for these students with consequent home-based, online and often sedentary lifestyles [7].

Existing literatures that investigated mental health conditions of the students during the pandemic were either conducted among the medical students, or included students from universities on the peripheries of Bangladesh. None discussed gender differences in terms of mental health disorders investigated [17–19].

Hence, this study aims to assess generalized anxiety disorder among university students during the COVID-19 pandemic using a gender lens. The findings can potentially foster the design of appropriate gender-based mental health interventions well-suited to educational institutions tailored to the special need of both men and women.

## Materials and methods

### Study design

A cross-sectional online survey was implemented among the students of two prominent public and private universities in Bangladesh (North South University and University of Dhaka) between May and August 2020. The research team included two of the faculties from both universities who played an instrumental role in developing a list of respondents. The list comprised email addresses and Facebook network accounts of potential respondents. The research team dispatched a web-based survey invitation to the students from list. In addition to this, the survey was also posted in the university associated Facebook groups as well as other smaller groups tied to the selected universities. The invitation contained a brief background, objectives, rights of the participants and the declaration of maintaining confidentiality. A contact address was also shared in the form, if any student had any queries before, during or after the survey. The students who accepted the invitation to participate in the survey browsed the invitation link and provided their responses.

### Sample size and data collection

**Fig. 1:**
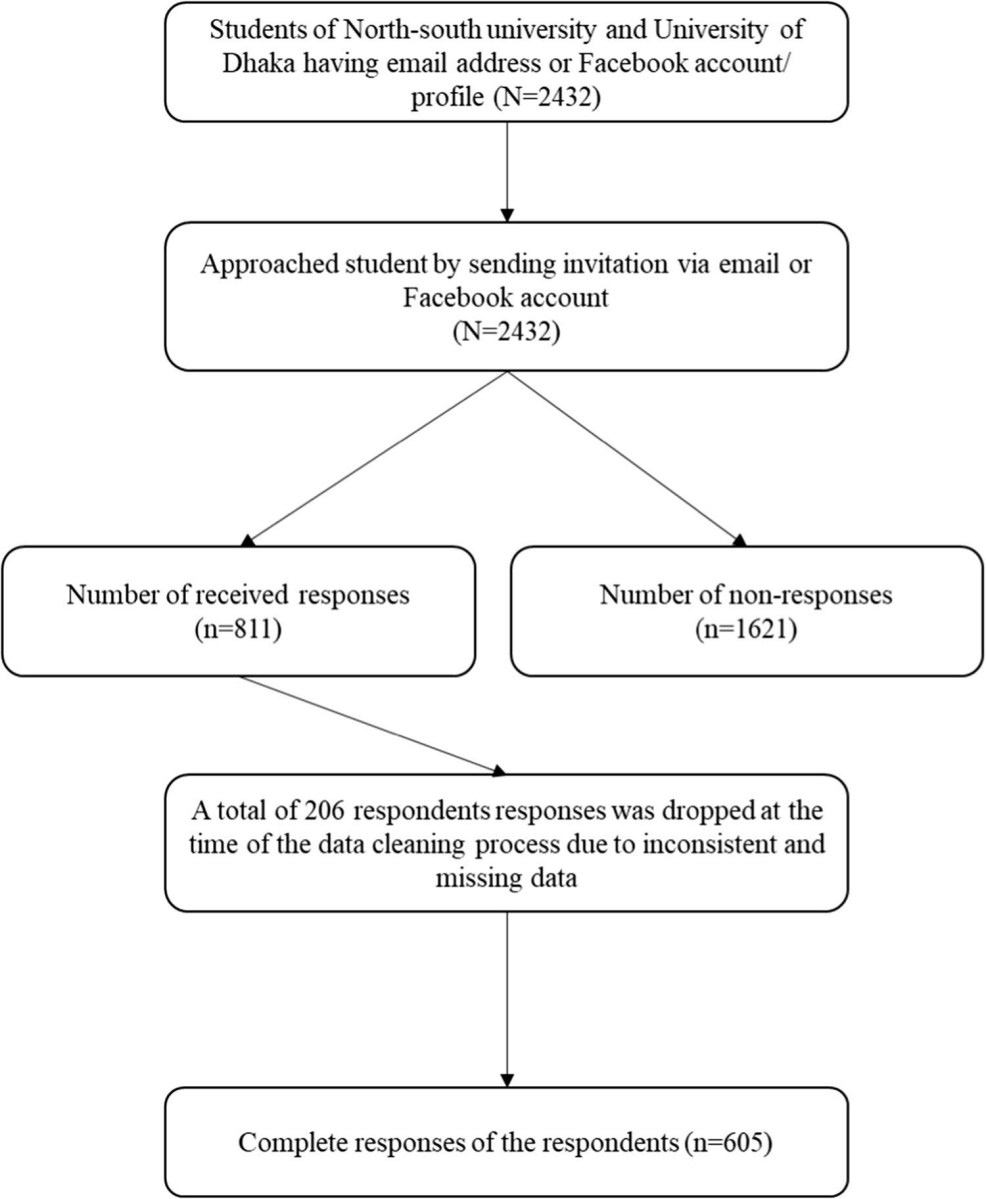
Data collection flow chart

The sample size was calculated using a single population proportion formula by considering the proportion of moderate to severe anxiety levels as 61.0% among university students in Bangladesh measured by GAD-7 [20]. In addition, assuming a 95% confidence level, 5% absolute precision, 10% non-response rates, and a 1.49-design effect, the estimated sample size is 605 [21]. Due to COVID-19 pandemic, government-imposed lockdown was going on during the data collection period. Consequently, our research team decided to conduct survey using online platform. The investigators were inclusive, open & circulate the form periodically for a maximum reach. Email addresses of the interested participants were collected to check their studentship for the reliability of the data. The research team approached 2432 students, and a total of 811 responses were received. We finally analyzed data from 605 respondents, excluding the others due to inconsistency and missing information. The data collection flow chart is shown in Fig. 1.

### Research tool

A self-reported, structured online questionnaire (both in Bangla and English) was distributed to the study participants along with an informed consent form. Each questionnaire had five dimensions: sociodemographic factors, questions on recreational activities, sleeping pattern, academic related information and the GAD-7 Scale. In the sociodemographic section, respondents were asked about age, gender, type of their residential areas, education level, marital status, occupation, number of family members, number of people within close circles infected by COVID-19, and feelings of distress due to the pandemic. Also, respondents were asked questions regarding their sleeping pattern and daily life activities such as sources of entertainment, feelings of concern evoked by reporting on the COVID-19 pandemic on television, levels of physical exercise, and engagement in household chores. Both English and Bangla versions of the seven-item generalized anxiety disorder (GAD-7) scale, validated in the Bangladeshi context, was used to identify the presence of anxiety among the participants [17, 22, 23]. Each item of the GAD-7 scale is scored on of a four-point Likert scale with; zero indicates no difficulty at all and three indicating extreme difficulty. Global summation of the scores results in a total between zero and 21, reflecting the severity of anxiety. A score of four or less indicated a minimal/no level of anxiety [24]. Scores between five and nine, inclusive, reflected mild anxiety levels, between 10 to 14, reflected moderate anxiety, and > 15 indicated severe anxiety [22]. Anxiety scores were then binarized based on a threshold score of 4 [24]. Participants with a GAD score less than or equal to 4 were deemed to have experienced no anxiety whilst the rest were considered to have experienced mild to severe levels of anxiety [24]. The study was conducted in line with the Checklist for Reporting Results of Internet E-Surveys (CHERRIES) guideline [25].

### Statistical analysis

The final dataset contained information of 605 students. For, further analysis of the data, we then used frequency and percentages for categorical variables, whilst, for continuous variables, mean and standard deviation (SD) were used to present descriptive statistics. We also conducted the chi-square test or fisher exact test (as appropriate) for categorical variables and Mann–Whitney test, Shapiro–Wilk test for the continuous variables to explore the significant gender-specific differences between gender-specific statistics and anxiety levels of the respondents. Multivariable logistic regressions were used to investigate gender-specific anxiety after controlling for socio-demographic factors, recreational activities, sleeping pattern, academic status [26]. In bivariate analysis, factors or variables with a p-value less than 0.05 were included in the multivariable regression model. The crude odds ratio (COR) for bivariable logistic models was reported with a 95% confidence interval, while the adjusted odds ratio (AOR) for multivariable logistic regression was reported with a 95% confidence interval. The Hosmer and Lemeshow goodness of fit tests were used to determine how well our model predicted the data. The Pseudo R-squares, such as the Cox and Snell's R-Square, the Nagelkerke's R-Square, and the McFadden's R-Square were utilized to indicate the percentage of variance in the outcome variable explained by the model. P-values less than 0.05 to indicate the statistical significance of the association. The analysis was performed using the SPSS software (version 25.0).

## Results

### Characteristics of the sample

Among the 605 study participants, 59.5% were female, where 40.5% were male. The mean age of the students was 23.1 years (SD ± 3.4). Most of them (96.4%) lived with their families during the COVID-19 pandemic, 90.9% were unmarried and 86.3% of students lived in urban areas (Table 1). The percentage of sleeping pattern that affected the daily life activities are similar among male (40%) and female (42%). A higher percentage of female students felt worried about academic delays than the males (65.3% vs 54.7%). The usage of Facebook as social media platform was higher among males (81.2%) than female users (71.4%) (Table 1).

**Table 1:** General Demographic information about the study participants, 2020 Dhaka Bangladesh

Variables	Female, n (%)	Male, n (%)	Total, n (%)
Total	360 (59.5)	245 (40.5)	605 (100)
Age in year			
Mean ± SD	23.3 ± 3.8	22.8 ± 2.8	23.1 ± 3.4
≤ 20	67 (18.61)	47 (19.18)	114 (18.84)
21–26	254 (70.56)	179 (73.06)	433 (71.57)
≥ 27	39 (10.83)	19 (7.76)	58 (9.59)
Mode of living			
Alone/ Hostel	6 (1.7)	16 (6.5)	22 (3.6)
Family	354 (98.3)	229 (93.5)	583 (96.4)
Type of residential area			
Rural	31 (8.6)	52 (21.2)	83 (13.7)
Urban	329 (91.4)	193 (78.8)	522 (86.3)
Educational institution			
Public university	155 (43.1)	135 (55.1)	290 (47.9)
Private university	205 (56.9)	110 (44.9)	315 (52.1)
Education level			
Undergraduate	252 (70)	179 (73.1)	431 (71.2)
Postgraduate	108 (30)	66 (26.9)	174 (28.8)
Year of study (only for undergraduate)			
1st year	30 (11.9)	26 (14.5)	56 (13)
2nd year	60 (23.8)	43 (24)	103 (23.9)
3rd year	60 (23.8)	50 (27.9)	110 (25.5)
Final year	102 (40.5)	60 (33.5)	162 (37.6)
Marital status			
Unmarried	316 (87.8)	234 (95.5)	550 (90.9)
Married	44 (12.2)	11 (4.5)	55 (9.1)
Current occupation			
Unemployed	38 (10.6)	31 (12.7)	69 (11.4)
Solely Student	252 (70)	168 (68.6)	420 (69.4)
Service	46 (12.8)	32 (13.1)	78 (12.9)
Business/Self-employed	24 (6.7)	14 (5.7)	38 (6.3)
Number of members in the family			
≤ 3	65 (18.10)	41 (16.7)	106 (17.5)
4–5	227 (63.00)	164 (66.9)	391 (64.6)
≥ 6	68 (18.90)	40 (16.4)	108 (17.9)
Close circle exposed by COVID-19			
Yes	223 (61.90)	131 (53.5)	354 (58.5)
No	122 (33.90)	101 (41.2)	223 (36.9)
No idea	15 (4.20)	13 (5.3)	28 (4.6)
Himself /herself infected with COVID-19			
Yes	23 (6.4)	9 (3.7)	32 (5.3)
No	337 (93.6)	236 (96.3)	573 (94.7)
Worried about the pandemic situation			
Yes	190 (52.8)	125 (51)	315 (52.1)
Sometimes	156 (43.3)	103 (42)	259 (42.8)
No	14 (3.9)	17 (7)	31 (5.1)
Way to keep on with social networking			
Facebook	257 (71.4)	199 (81.2)	456 (75.4)
I don't feel like to contact anyone	41 (11.4)	15 (6.1)	56 (9.3)
Phone calls (either video/audio)	62 (17.2)	31 (12.7)	93 (15.4)
Feeling worried about COVID-19 news on television			
Yes	168 (46.7)	122 (49.8)	290 (47.9)
No	192 (53.3)	123 (50.2)	315 (52.1)
Maintaining a regular physical exercise			
Yes	57 (15.8)	54 (22)	111 (18.3)
Occasionally	170 (47.2)	88 (35.9)	258 (42.6)
No	133 (36.9)	103 (42)	236 (39)
Doing household chores			
Yes	209 (58.1)	100 (40.8)	309 (51.1)
Occasionally	120 (33.3)	96 (39.2)	216 (35.7)
No	31 (8.6)	49 (20)	80 (13.2)
Feeling any difficulty in sleeping at night			
Severe	127 (35.3)	67 (27.4)	194 (32.1)
Moderate	94 (26.1)	75 (30.6)	169 (27.9)
Mild	139 (38.6)	103 (42)	242 (40)
Stressed about the sleeping pattern			
Always	97 (26.9)	59 (24.1)	156 (25.8)
Often	52 (14.4)	33 (13.5)	85 (14)
Sometime	144 (40)	92 (37.6)	236 (39)
Not at all	67 (18.6)	61 (24.9)	128 (21.2)
Current sleeping pattern affecting daily life			
Always	150 (41.7)	98 (40)	248 (41)
Often	9 (2.5)	10 (4.1)	19 (3.1)
Sometime	148 (41.1)	88 (35.9)	236 (39)
Not at all	53 (14.7)	49 (20)	102 (16.9)
Worried about academic delays			
Yes	235 (65.3)	134 (54.7)	369 (61)
Sometimes	82 (22.8)	53 (21.6)	135 (22.3)
No	43 (11.9)	58 (23.7)	101 (16.7)
Have any pre-existing psychological issues			
Yes	82 (22.8)	46 (18.8)	128 (21.2)
I feel like but not sure about it	124 (34.4)	70 (28.6)	194 (32.1)
No	154 (42.8)	129 (52.7)	283 (46.8)
Proportion of mild to severe level of anxiety			
Yes	327 (90.80)	202 (82.40)	529 (87.40)
No	33 (9.20)	43 (17.50)	76 (12.60)

### Prevalence of gender-specific anxiety disorder

**Fig. 2:**
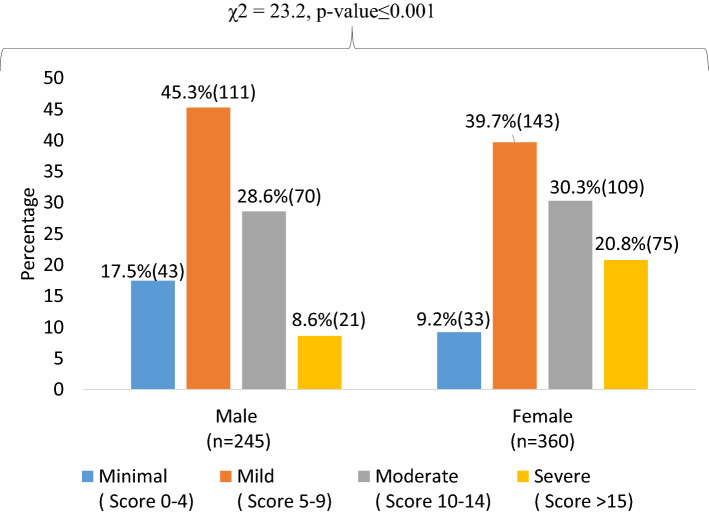
Percentage distribution of severity level of anxiety among students

The overall mean of GAD-7 score of the participants was 9.6 (SD = 4.6); among women it was 10.3 (SD = 4.8); whereas it was 8.4 (SD = 4.2) for men. The distribution of women across the different severities of anxiety was as follows: minimum (9.2%), mild (39.7%), moderate (30.3%) and severe (20.8%). On the other hand, about 17.5%, 45.3%, 28.6%, and 8.6% of male students were suffering from minimum, mild, moderate and severe anxiety respectively (Fig. 2).

We found that 529 of 605 student participants reported a mild to severe degree of anxiety, with women accounting for 61.8% (n = 327) and men accounting for 38.2% (n = 202) (Table 1). The prevalence of anxiety among women participants who were from private university, unmarried, staying with family and in urban areas were respectively 57.8%, 87.8%, 98.2% and 91.1%, and for men, these were 43.1%, 97.0%, 93.6% and 77.7% respectively. The prevalence of anxiety was 45.6% and 67.3%, among the women who reported an interruption in daily life activities due to sleeping patterns and worry about academic delays. Whereas for the men, the prevalence was 42.1% and 59.4%, respectively (Table 2).

**Table 2:** Prevalence of mild to severe levels of anxiety risks measured by GAD-7 among the male and female students, during COVID-19 pandemic according to their characteristics, 2020 Dhaka Bangladesh

Variables	Without anxiety (minimal/normal) (N = 76)		With anxiety (mild to severe) (N = 529)		
	Female (N = 33)	Male (N = 43)	Female (N = 327)	Male (N = 202)	p-value
	n (%)	n (%)	n (%)	n (%)	
Age in year					
Mean ± SD	25.03 ± 3.90	23.86 ± 3.68	23.15 ± 3.79	22.62 ± 2.54	
≤ 20	0 (0.00)	6 (13.95)	67 (20.49)	41 (20.30)	0.580
21–26	25 (75.76)	32 (74.42)	229 (70.03)	147 (72.77)	
≥ 27	8 (24.24)	5 (11.63)	31 (9.48)	14 (6.93)	
Mode of living					
Alone/Hostel	0 (0)	3 (6.98)	6 (1.83)	13 (6.44)	0.006
Family	33 (100.0)	40 (93.02)	321 (98.17)	189 (93.56)	
Type of residential area					
Rural	2 (6.06)	7 (16.28)	29 (8.87)	45 (22.28)	< 0.001
Urban	31 (93.94)	36 (83.72)	298 (91.13)	157 (77.72)	
Educational institution					
Public university	17 (51.52)	20 (46.51)	138 (42.20)	115 (56.93)	0.001
Private university	16 (48.48)	23 (53.49)	189 (57.80)	87 (43.07)	
Education level					
Undergraduate	17 (51.52)	26 (60.47)	235 (71.87)	153 (75.74)	0.327
Postgraduate	16 (48.48)	17 (39.53)	92 (28.13)	49 (24.26)	
Year of study (only for undergraduate)					
1st year	1 (5.88)	4 (15.38)	29 (12.34)	22 (14.38)	0.516
2nd year	1 (5.88)	6 (23.08)	59 (25.11)	37 (24.18)	
3rd year	3 (17.65)	5 (19.23)	57 (24.26)	45 (29.41)	
Final year	12 (70.59)	11 (42.31)	90 (28.30)	49 (32.03)	
Marital status					
Unmarried	29 (87.88)	38 (88.37)	287 (87.77)	196 (97.03)	< 0.001
Married	4 (12.12)	5 (11.63)	40 (12.23)	6 (2.97)	
Current occupation					
Unemployed	4 (12.12)	8 (18.60)	34 (10.40)	23 (11.39)	0.889
Solely Student	22 (66.67)	24 (55.81)	230 (70.34)	144 (71.29)	
Service	6 (18.18)	8 (18.60)	40 (12.23)	24 (11.88)	
Business/Self-employed	1 (3.03)	3 (6.98)	23 (7.03)	11 (5.45)	
Number of family members					
≤ 3	6 (18.18)	7 (16.28)	59 (18.04)	34 (16.83)	0.223
4–5	20 (60.61)	23 (53.49)	207(60.30)	141 (69.80)	
≥ 6	7 (21.21)	13 (30.23)	61 (18.65)	27 (13.37)	
Close circle exposed by COVID-19					
No	12 (36.36)	23 (53.49)	110 (33.64)	78 (38.61)	0.365
Yes	20 (60.61)	18 (41.86)	203 (62.08)	113 (55.94)	
No idea	1 (3.03)	2 (4.65)	14 (4.28)	11 (5.45)	
Himself /herself infected with COVID-19					
No	33 (100.0)	41 (95.35)	304 (92.97)	195 (96.53)	0.085
Yes	0 (0)	2 (4.65)	23 (7.03)	7 (3.47)	
Worried about the pandemic situation					
No	4 (12.12)	5 (11.63)	10 (3.06)	12 (5.94)	0.237
Sometimes	20 (60.61)	26 (60.47)	136 (41.59)	77 (38.12)	
Yes	9 (27.27)	12 (27.91)	181 (55.35)	113 (55.94)	
Way to keep on with social networking					
Facebook	23 (69.70)	32 (74.42)	234 (71.56)	167 (82.67)	0.010
I don't feel like to contact anyone	3 (9.09)	4 (9.30)	38 (11.62)	11 (5.45)	
Phone calls (either video/audio)	7 (21.12)	7 (16.28)	55 (16.82)	24 (11.88)	
Feeling worried about COVID-19 news on television					
No	22 (66.67)	30 (69.77)	170 (51.99)	93 (46.04)	0.184
Yes	11 (33.33)	13 (30.23)	157 (48.01)	109 (53.96)	
Maintaining a regular physical exercise					
No	8 (24.24)	16 (37.21)	125 (38.23)	87 (43.07)	0.078
Sometimes	14 (42.42)	11 (25.58)	156 (47.71)	77 (38.12)	
Yes	11 (33.33)	16 (37.21)	46 (14.07)	38 (18.81)	
Doing household chores					
No	3 (9.09)	8 (18.60)	28 (8.56)	41 (20.30)	< 0.001
Sometimes	8 (24.24)	17 (39.53)	112 (34.25)	79 (39.11)	
Yes	22 (66.67)	18 (41.86)	187 (57.19)	82 (40.59)	
Feeling any difficulty in sleeping at night					
Severe	2 (6.06)	9 (20.93)	125 (38.23)	58(28.71)	0.082
Moderate	6 (18.18)	12 (27.91)	88 (26.91)	63 (31.19)	
Mild	25 (75.76)	22 (51.16)	114 (34.86)	81 (40.10)	
Stressed about the sleeping pattern					
Always	0 (0.00)	5 (11.63)	97 (29.66)	54 (26.73)	0.420
Often	4 (12.12)	2 (4.65)	48 (14.68)	31 (15.35)	
Sometimes	8 (24.24)	14 (32.56)	136 (41.59)	78 (38.61)	
Not at all	21 (63.64)	22 (51.16)	46 (14.07)	39 (19.31)	
Current sleeping pattern affecting daily life					
Always	1 (3.03)	13 (30.23)	149 (45.57)	85 (42.08)	0.241
Often	1(3.03)	3 (6.98)	8 (2.45)	7 (3.47)	
Sometimes	13 (39.39)	11 (25.58)	135 (41.28)	77 (37.12)	
Not at all	18 (54.55)	16 (37.21)	35 (10.70)	33 (16.34)	
Worried about academic delays					
Yes	15 (45.45)	14 (32.56)	220 (67.28)	120 (59.41)	0.001
Sometimes	9 (27.27)	16 (37.21)	73 (22.32)	37 (18.32)	
No	9 (27.27)	13 (30.23)	34 (10.40)	45 (22.28)	
Have any pre-existing psychological issues					
No	27 (81.82)	35 (81.40)	127 (38.84)	94 (46.53)	0.2
Yes	1 (3.03)	5 (11.63)	81 (24.77)	41 (20.30)	
I feel like but not sure about it	5 (15.15)	3 (6.98)	119 (36.39)	67 (33.17)	

### Factors associated with gender difference in the prevalence of anxiety

Table 3 represents the odds ratios from the logistic regression analysis. The multivariable logistic regression model included age in years, sex, type of residential area, mode of living, educational institution, and marital status as covariates. These covariates were found to be statistically significant in a bivariable analysis. We found in the adjusted model that age in years, sex and worry about academic delays were significantly associated with mild to severe levels of anxiety (Table 3). Female university students were 2.21 times more likely [AOR: 2.21; CI 95% (1.28–53.70); p-value: 0.004] to report anxiety than male students. Likewise, participants who reported worrying about their academic delays had about 2.82 times greater odds of anxiety compared to the counter reference category. Other than that, lower probabilities of anxiety were found among students aged 21–26 years and more than or equal to 27.

**Table 3:** Multiple logistic regression analysis of the male and female students during COVID-19 pandemic with their characteristics, 2020 Dhaka Bangladesh

Variables	Anxiety (mild to severe levels)			
	COR (95% CI)	p-value	AOR (95% CI)	p-value
Age in year				
21–26	0.37 (0.15–0.87)	0.023	0.34 (0.14–0.84)	0.019
≥ 27	0.19 (0.07–0.54)	0.002	0.17 (0.05–0.57)	0.001
≤ 20	Reference			
Sex				
Female	2.11 (1.29–3.43)	0.003	2.21 (1.28–3.7)	0.004
Male	Reference		Reference	
Type of residential area				
Urban	1.21 (0.58–2.53)	0.611	0.68 (0.31–1.50)	0.343
Rural	Reference		Reference	
Mode of living				
Family	1.10 (0.32–3.82)	0.877	0.78 (0.0.20–3.02)	0.722
Alone/Hostel	Reference		Reference	
Educational institution				
Public university	1.10 (0.32–3.82)	0.889	0.84 (0.50–1.43)	0.533
Private university	Reference		Reference	
Marital status				
Married	0.71 (0.33–1.51)	0.374	1.14 (0.0.42–3.1)	0.797
Unmarried	Reference		Reference	
Way to keep on with social networking				
Facebook	1.29 (0.69–2.44)	0.429	1.34 (0.68–2.64)	0.39
I don't feel like to contact anyone	1.24 (0.47–3.29)	0.665	1.03 (0.37–2.87)	0.955
Phone calls (either video/audio)	Reference		Reference	
Doing household chores				
Yes	0.93 (0.46–1.91)	0.849	0.89 (0.41–1.94)	0.773
Occasionally	1.14(0.67–1.94)	0.639	1.03 (0.46–2.32)	0.929
No	Reference		Reference	
Worried about academic delays				
Yes	3.27 (1.78–5.98)	< 0.001	2.82 (1.50–5.31)	0.001
Sometimes	1.23 (0.65–2.33)	0.535	1.07 (0.54–2.09)	0.844
No	Reference			

COR: Crude odds ratio; AOR: Adjusted odds ratio; CI: Confidence Interval

Hosmer–Lemeshow tests: Chi-square (p-value) = 7.11 (0.5246)

Cox and Snell's R-Square = 0.063

Nagelkerke's R-Square = 0.119

McFadden's R-Square = 0.086

## Discussion

Evidence shows that major pandemic or epidemic outbreaks have always been associated with a spike in mental health consequences which usually deteriorates further as the outbreaks progress [17, 18, 27–30]. Similarly, countries worldwide have been affected much with multiple waves of the COVID-19 pandemic and each wave has depicted different trajectories of daily new COVID-19 cases, transmission and mortality rates [31]. To date, Bangladesh has experienced two waves of the pandemic with the second wave being more devastating [32]. Aligning with this, prior studies have reported of declining mental health conditions during the second wave of COVID-19 compared to the first wave of COVID-19 [29, 33]. However, it also has to be considered that the first wave of COVID-19 did create immense tension among the population due to sudden outbreak of a new disease leading to strict lockdown, uncertainties of the infection, confinement of social spaces and management of professional duties [34]. Hence, our study conducted was during the first wave of the pandemic to assess the mental health conditions among the university students in Bangladesh during this period.

Furthermore, in prior studies, gender disparities also existed among the prevalence of mental health conditions. Similarly in Bangladesh, gender differences were significantly associated with increased anxiety especially among women and students during both the waves of pandemic [27, 35]. Additionally in a prior study, gender and its dependence on cross-cultural differences has essentially influenced the prevalence of mental health conditions during this pandemic [36]. With very limited number of studies using a gender specific lens. This is one of the very few studies that has examined student mental health from urban areas involving two of the largest universities of Bangladesh through a gender-specific lens. By analyzing anxiety disorders among students during the first wave of pandemic through a gender-specific lens, this study will support in devising comprehensive interventions in case of future emergencies.

From the pre-COVID-19 period, university students from Bangladesh were reported to have higher levels of common mental health issues than other population groups [17, 19, 23]. Furthermore, in comparison to pre-COVID-19 era, possible factors such as fear of infection, fabricated news, and academic delay, social and financial insecurities led to a rise in mental health conditions among university students during the pandemic [6, 16, 17, 37–41]. Likewise, our study also revealed a higher over-all prevalence of mild to severe generalized anxiety disorder among women 61.8% (N = 327) relative to men 38.2% (N = 202) within the university students population during this pandemic.

Concurrent evidence from LMICs has affirmed that the prescribed gender roles have resulted in young females being burdened with domestic duties, financial dependency, lesser coping strategies, and more uncertainty during the lockdown, making them prone to developing mental health symptoms [18, 30, 42–45]. A plethora of studies from worldwide and Bangladesh, supported the idea that the prevalence of anxiety is higher among female students than males during the pandemic [6, 16–18, 37–41, 44, 46–50]. However, no statistical association between gender and psychological impact was explored and reported in several other studies from both Southern and Southeast Asian countries [20, 51–53].

In multivariate analysis, our study reports that, age in years and being worried about academic delays were significantly associated with the level of anxiety. However, our study also revealed that anxiety and age are associated negatively among the women. Similar findings were reported in other LMICs where younger and newly enrolled students are already under the pressure of academic competition in a new environment, the lockdown has limited their scope of communication to build an inter-personal relationship with academic departments which maybe some of the causes of anxiety at a younger age [54–56]. Whereas, some prior studies also reported of age being a positive factor for mental health conditions among students [54–57].

Due to lockdown related interruptions in routine teaching and assessments, the possibilities for the academic delay exist that can also jeopardise the expected graduation time of the students. In our study, participants who were worried about academic delays were significantly more likely to be anxious (AOR2.21 than who were not worried about it (*p*-value: 0.004). It has to be realized that the current pandemic has added uncertainties for the graduating students in finding jobs and enrolling in further education or may be finding internships [17]. Moreover, in South Asian countries like Bangladesh, women already have shorter time frame available for career development and marriage, the academic delay owing to the pandemic has further worsened scope thus leading to anxiety [17, 54, 57, 58]. Studies both from global and South Asian countries also reported that students especially from the final years or the ones nearing graduation were found to be effected psychologically due to worrying about their academic curriculum [17, 58, 59]. As per prior studies, it can be concluded that women with education attainment are in socio-economically empowered position, which significantly reduces their risk of being mentally vulnerable [60–62]. Hence, it can be assumed that academic delays reduce the chances of women to be financially independent and empowered which in turn affects their mental health condition.

Also, in bivariate analysis factors like studying in private university, being unmarried, staying with the family, urban area residence, interrupted daily life activities due to sleeping pattern and worrying about academic delays contributed to higher prevalence of anxiety among females students. However, the findings were found to be non-significant while adjusting the confounding factors, which might be due to the limited sample size.

So, our study findings align with the fact that gender stereotypes have reinforced social stigma and constrained mental health help-seeking behavior in both men and women. Moreover, viewing mental health through the gender specific lens is crucial, especially for the female of developing countries. So, overlooking this bias among the youth could have drastic consequences on the national priority and productivity as the youth plays an important role in the country's economic growth. In addition, the study findings can be used to design need based and gender specific interventions for an effective mental health outcome among the youth population in Bangladesh.

## Strength and limitations

Nevertheless, many limitations of the current study have been determined by the investigators. This was an online based survey therefore, there is no chance of observation and validation of the data given by the students. The limited sample size limits the generalizability of the findings. The identification of anxiety was entirely subjective; we did not apply the tools designed especially for the COVID-19 pandemic, for instance, the Coronavirus Anxiety Scale (CAS) [63]. The study also cannot establish the causal factors as it is a cross-sectional study. Self-reported measures may be responsible for recall and social desirability biases the present study does not provide any in-depth information behind the gender differences on the anxiety level among the students. Therefore, a qualitative analysis is highly recommended among the university students. In addition, there is also a possibility for selection bias associated with data collection method, as the survey could have attracted more students who either are suffering or free from anxiety disorder. Either way, this selection bias could have either inflated or underestimated the assessed prevalence.

However, despite all these limitations, the current study used the gender specific approach that was not used in any previously published studies focusing on mental health and COVID-19 among the students. The results showing that almost twice the number of female students were suffering from anxiety than male students suggesting that female student’s mental health status should be monitored regularly. They might need holistic psychosocial interventions targeting women as a larger part of society, multi-disciplinary interventions involving primary care, judicial and legal fronts, and interventions. In addition, on comparing the impact of lockdown due to COVID-19 on the mental health status of students from private and public universities suggested that private university students might need additional psycho-social support during this pandemic. Thus, the current study viewed the student’s mental health status via the gender lens, which was much needed to identify the vulnerable population group and design a gender-specific, social and policy level psychosocial interventions.

## Conclusion

The COVID-19 pandemic has severe repercussion on mental health, especially among the young female university students in Bangladesh. Gender-specific and campus-based-low-intensity psychosocial interventions are needed to ensure effective mental health services for Bangladeshi university students. Besides, a nationwide longitudinal survey needs to be conducted to generate robust evidence that will sensitize the policymakers to formalize an urgent, feasible, and sustainable intervention model for an emergency condition such as a pandemic situation. Moreover, this study also creates the scope of comparative studies and doing further large-scale longitudinal studies assessing the mental health conditions considering the outbreak of new variants at different time points with a gender lens.

## Data Availability

The datasets generated and/or analyzed during the current study are available from the corresponding author on reasonable request.
